# *In Vivo* Micro-CT Assessment of Airway Remodeling in a Flexible OVA-Sensitized Murine Model of Asthma

**DOI:** 10.1371/journal.pone.0048493

**Published:** 2012-10-30

**Authors:** Mathieu Lederlin, Annaïg Ozier, Gaël Dournes, Olga Ousova, Pierre-Olivier Girodet, Hugues Begueret, Roger Marthan, Michel Montaudon, François Laurent, Patrick Berger

**Affiliations:** 1 Univ. Bordeaux, Centre de Recherche Cardio-thoracique de Bordeaux, U1045, CIC 0005, F-33000 Bordeaux, France; 2 INSERM, Centre de Recherche Cardio-thoracique de Bordeaux, U1045, CIC 0005, F-33000 Bordeaux, France; 3 CHU de Bordeaux, Service d’Imagerie Thoracique et Cardiovasculaire, Service d’Exploration Fonctionnelle Respiratoire, CIC 0005, Service d’Anatomopathologie, F-33604 Pessac, France; Charite Universitätsmedizin, Germany

## Abstract

Airway remodeling is a major pathological feature of asthma. Up to now, its quantification still requires invasive methods. In this study, we aimed at determining whether *in vivo* micro-computed tomography (micro-CT) is able to demonstrate allergen-induced airway remodeling in a flexible mouse model of asthma. Sixty Balb/c mice were challenged intranasally with ovalbumin or saline at 3 different endpoints (Days 35, 75, and 110). All mice underwent plethysmography at baseline and just prior to respiratory-gated micro-CT. Mice were then sacrificed to assess bronchoalveolar lavage and lung histology. From micro-CT images (voxel size = 46×46×46 µm), the numerical values of total lung attenuation, peribronchial attenuation (PBA), and PBA normalized by total lung attenuation were extracted. Each parameter was compared between OVA and control mice and correlation coefficients were calculated between micro-CT and histological data. As compared to control animals, ovalbumin-sensitized mice exhibited inflammation alone (Day 35), remodeling alone (Day 110) or both inflammation and remodeling (Day 75). Normalized PBA was significantly greater in mice exhibiting bronchial remodeling either alone or in combination with inflammation. Normalized PBA correlated with various remodeling markers such as bronchial smooth muscle size or peribronchial fibrosis. These findings suggest that micro-CT may help monitor remodeling non-invasively in asthmatic mice when testing new drugs targeting airway remodeling in pre-clinical studies.

## Introduction

Airway inflammation and remodeling are well-established features of asthma even if their complex relationships are not fully understood [1], [2]. Airway remodeling refers to structural changes such as bronchial fibrosis, increase in basal membrane thickness and smooth muscle size [3]. In particular, smooth muscle remodeling has been associated with a decrease in lung function leading to a more severe asthma phenotype [4], [5]. Moreover, recent advance of new therapies targeting remodeling, either in human asthma [6], [7] or in mouse model of asthma [8], [9], has made it critical to develop non-invasive tools for assessing remodeling. Currently, histology is still the standard method for identifying and grading airway remodeling but its use is limited by its invasiveness. By contrast, imaging techniques such as thin-section computed tomography (CT) are non-invasive and have been shown to identify asthma-related structural changes [10]–[14], without distinguishing however, between inflammation and remodeling.

Research on animal models of human diseases is of main importance for filling the gap between fundamental concepts and their clinical applications. In this way, imaging techniques in animals should strive to target as specific pathological processes as possible, *i.e*. inflammation and remodeling in the case of asthma. Moreover, from a translational viewpoint, imaging of animals should ideally be *in vivo*, thereby allowing longitudinal cohort studies and follow-up of new therapeutic effects [15]. *In vivo* micro-computed tomography (micro-CT) has been shown to be promising by demonstrating peribronchial changes in an ovalbumin-sensitized mouse asthma model [16]. In this latter study, the peribronchial attenuation value extracted from micro-CT images was significantly increased in sensitized mice as compared to control mice and was correlated with some remodeling components such as bronchial smooth muscle size. However, both inflammation and remodeling were present in this model and could account for the increased peribronchial attenuation. Moreover, inflammation spread over the boundaries of the bronchial wall within the lung parenchyma [16], [17] and could alter total lung attenuation. We thus hypothesized that the normalization of the peribronchial attenuation by the total lung attenuation could be more specific to assess bronchial remodeling.

The aims of our study were then (i) to develop a flexible mouse model of allergic asthma exhibiting inflammation alone, remodeling alone, or both characteristics together, (ii) to validate a semi-automatic method enabling a quick and reproducible assessment of peribronchial attenuation and total lung attenuation from micro-CT datasets, and (iii) to determine whether the peribronchial attenuation or the normalized peribronchial attenuation could be related to airway remodeling.

## Materials and Methods

### Animals

Sixty female BALB/c mice (5 weeks old) were purchased from Elevage Janvier (Le Genest-Saint-Isle, France) and acclimatised in environmentally controlled conditions for 1 week prior to study and for the duration of the experiments. All animal use procedures were approved by our local Animal Care Committee. This study complied with the European law and the Guide for the Care and Use of Laboratory Animals of the National Institutes of Health.

**Figure 1:**
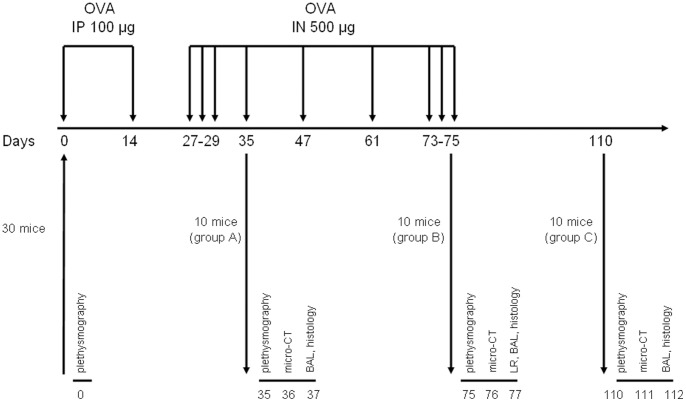
Chronologic diagram of OVA-sensitized in mice. Three groups of mice are generated: group A (days 35 to 37), group B (days 75 to 77), group C (days 110 to 112). OVA =  ovalbumin, IP =  intraperitoneal, IN = intranasal, BAL =  bronchoalveolar lavage, LR = lung resistance.

**Figure 2:**
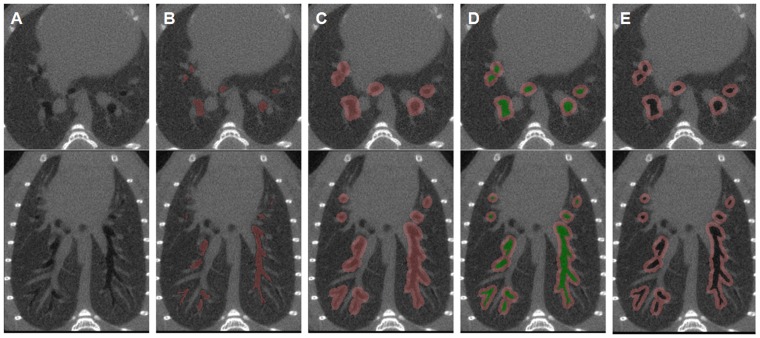
Semi-automatic 3D method for assessing peribronchial attenuation. A) Native axial (top) and coronal thin-section reformatted (bottom) micro-CT images of the bronchial tree. B) Automatic segmentation of the bronchial lumen (pink). C) Automatic 8-voxels dilatation of the lumen volume. D) Second automatic segmentation of the bronchial lumen volume (green) overwriting bronchial lumen from the previous volume of interest. E) After subtraction of the bronchial lumen, the resultant volume of interest includes only the peribronchial area of the whole bronchial tree. From the created peribronchial volume, the software provides the peribronchial mean attenuation (PBA) value.

**Table 1:** Description of the 3 asthmatic mouse models.

	Group A (Days 35–37)			Group B (Days 75–77)			Group C (Days 110–112)		
	Control(n = 8)	OVA(n = 8)	P-value	Control(n = 9)	OVA(n = 10)	P-value	Control(n = 8)	OVA(n = 8)	P-value
**Weight at endpoint (g)**	18.4±0.3	18.9±0.4	0.234	22.33±0.4	21.1±0.6	0.150	24.3±0.8	23.4±0.5	0.450
**Plethysmography**									
Penh ratio at baseline	2.3±0.3	2.4±0.3	0.916	2.5±0.3	2.0±0.2	0.156	2.3±0.4	2.4±0.3	0.959
Penh ratio at endpoint	3.5±0.5	10.0±3.6	0.001	3.4±0.8	7.5±1.4	0.045	2.8±0.3	3.5±0.6	0.279
**BAL**									
Total cells (× 10^4^/mL)	36.4±7.5	48.6±6.8	0.105	35.6±2.3	76.9±12.8	<0.001	24.0±2.4	36.0±5.8	0.172
% macrophages	93.3±2.1	86.4±2.8	0.050	91.8±0.6	60.4±9	0.013	95.8±1.3	93.7±1.6	0.382
% neutrophils	3.3±1.2	3.2±2.8	0.125	3.2±1.2	18±7	0.573	0.8±0.1	1.5±0.7	0.421
% eosinophils	0.6±0.6	4.2±0.9	0.003	0.4±0.4	10.6±3.1	0.002	0.0±0.0	0.1±0.1	0.160
% lymphocytes	2.8±1.4	6.3±1.6	0.038	4.7±0.9	11±2.1	0.081	3.4±1.2	4.6±1.7	0.878
**Histological data**									
Peribronchial space area(µm^2^)	14.5±2.2	25.6±5.1	0.083	13.6±1.2	56.2±10.2	<0.001	11.3±1.3	14.4±1.43	0.083
Number of nucleated cellswithin the peribronchialspace area (cell/µm^2^)	106.8±11.9	171.2±28.4	0.028	138.6±19.2	505±85	<0.001	76.7±7.1	102.3±9.7	0.028
Basal membrane thickness(µm)	5.4±0.3	5.9±0.2	0.065	5.3±0.4	10.2±0.9	<0.001	5.5±0.3	8.9±0.6	<0.001
Wall area (µm^2^/bronchus)	17.4±2.4	18.4±1.4	0.442	16.7±1.0	25.4±1.6	<0.001	16.2±0.7	24.5±2.4	<0.001
Bronchial muscle area(× 10^3^ µm^2^/bronchus)	4.3±0.9	3.6±0.5	0.798	4.1±0.8	6.6±0.9	0.035	3.6±0.4	6.9±0.6	0.002
Peribronchial fibrosis(× 10^3^ µm^2^)	1.9±0.6	2.8±0.4	0.105	2.6±0.4	6.1±1.1	0.012	2.0±0.3	5.6±1.2	0.005

Data are means ± standard error of the mean. P-values were obtained using Wilcoxon-Mann-Whitney rank sum test. BAL: bronchoalveolar lavage.

### Models of Allergic Asthma and Scheme of the Study

The challenge protocols were modified from that described previously [16]. Thirty mice were sensitized by two intraperitoneal injections of 100 µg of ovalbumin (OVA) on days 0 and 14 in the absence of aluminium hydroxide. All the mice were anaesthetised using an intraperitoneal injection of both 50 µg/g ketamine (Panpharma, Fougeres, France) and 5 µg/g xylazine (Sigma-Aldrich, Saint-Quentin-Fallavier, France). They were then challenged intranasally with 500 µg of OVA at different days (Figure 1). Three different endpoints were used to obtain 3 groups of 10 mice: group A was analyzed at days 35–37, group B was analyzed at days 75–77, and group C was analyzed at days 110–112. Thirty other mice received normal saline intraperitoneally and intranasally on the same days and constitute 3 control groups corresponding to the 3 various endpoints. This study complied with the European law and the Guide for the Care and Use of Laboratory Animals of the National Institutes of Health.

### Plethysmography

Bronchial hyperresponsiveness (BHR) to methacholine (Sigma-Aldrich, Saint-Quentin Fallavier, France) was measured in both unrestrained conscious mice by single-chamber plethysmography at baseline and at each endpoint, and in anesthetized mice by invasive plethysmography at Day 77 (Emka Technologies, Paris, France). Enhanced pause parameter (Penh) was measured in unrestrained conscious mice whereas lung resistance (LR) was measured in anesthetized mice. Results were averaged for 3 min, 30 s after each successive inhalation of an increasing dose of aerosolised methacholine (1–16 mg/ml) [16]. The results were expressed as a ratio of Penh or LR as a ratio of values measured in response to methacholine (8 mg/ml) to that with normal saline. Ratios of Penh measured at Day 75 were compared to that of LR measured at Day 77 in both OVA and control animals.

**Figure 3:**
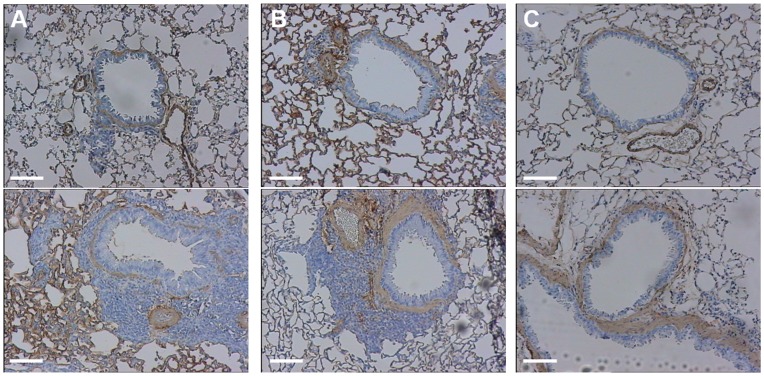
Representative optic microscopic images (100× magnification) from bronchial sections stained with anti-α-smooth muscle actin antibody obtained from control (top) and OVA-sensitized mice (bottom). Bars represent 85 µm. A) mice from group A. B) mice from group B. C) mice from group C. There is a diffuse cell infiltration of the peribronchial space in OVA-sensitized mice from groups A and B, as compared to OVA-sensitized mice from group C (nuclei are stained in dark blue). OVA-sensitized mice from groups B and C exhibit an increased smooth-muscle mass (alpha-actin is stained in brown), as compared to OVA-sensitized mice from group A.

**Figure 4:**
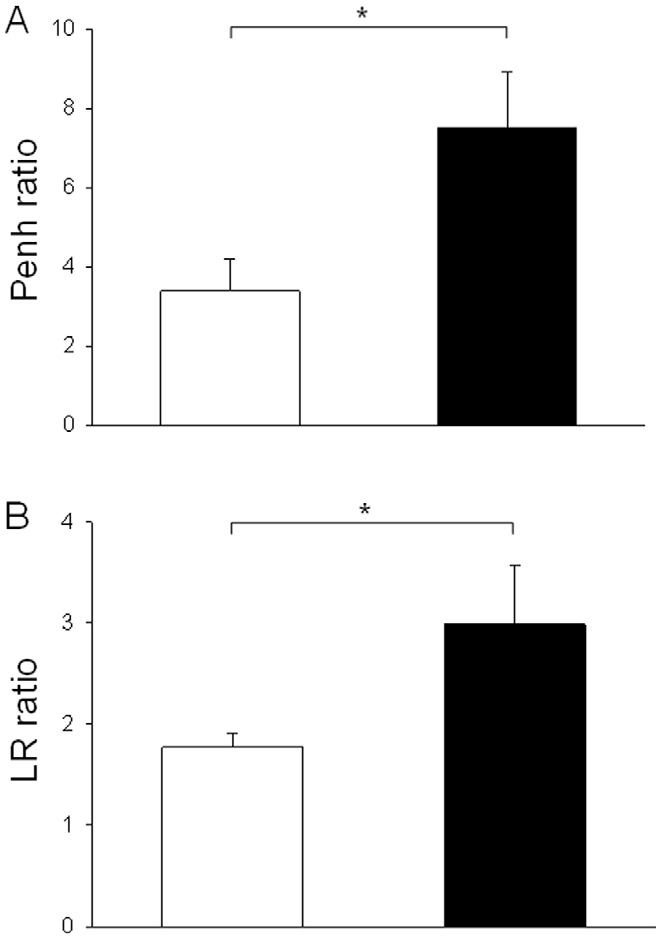
Comparison of Penh and lung resistance. A) Bronchial hyperresponsiveness (BHR) to methacholine was determined at Day 75 in unrestrained conscious mice by single-chamber plethysmography. The results were expressed as a ratio of Penh measured in response to 8 mg/ml methacholine to that with normal saline. B) Bronchial hyperresponsiveness (BHR) to methacholine was also determined at Day 77 in anaesthetised and intubated animals by invasive plethysmography. The results were expressed as a ratio of LR measured in response to 8 mg/ml methacholine to that with normal saline. Results from control (white bars) and OVA-sensitized mice (black bars) are presented.

**Figure 5:**
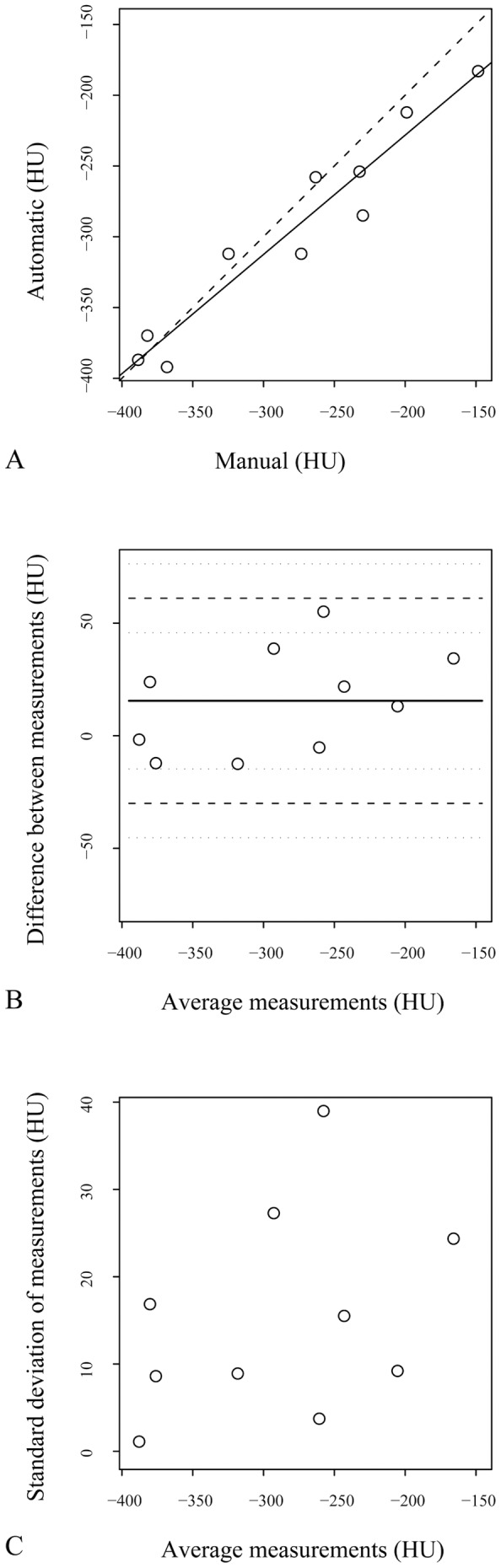
Bland-Altman analysis of manual and semi-automatic methods for peribronchial attenuation (PBA) measurements. A) Correlation of peribronchial mean attenuation (PBA) between the two methods. Dashed line represents the line of equality. Solid line corresponds to the regression line. B) Means of measurement between the two methods are plotted against their differences. Solid line corresponds to the mean difference. Dashed lines correspond to the mean difference ±2 standard deviations. C) Means of measurement between the two methods are plotted against their standard deviations.

**Figure 6:**
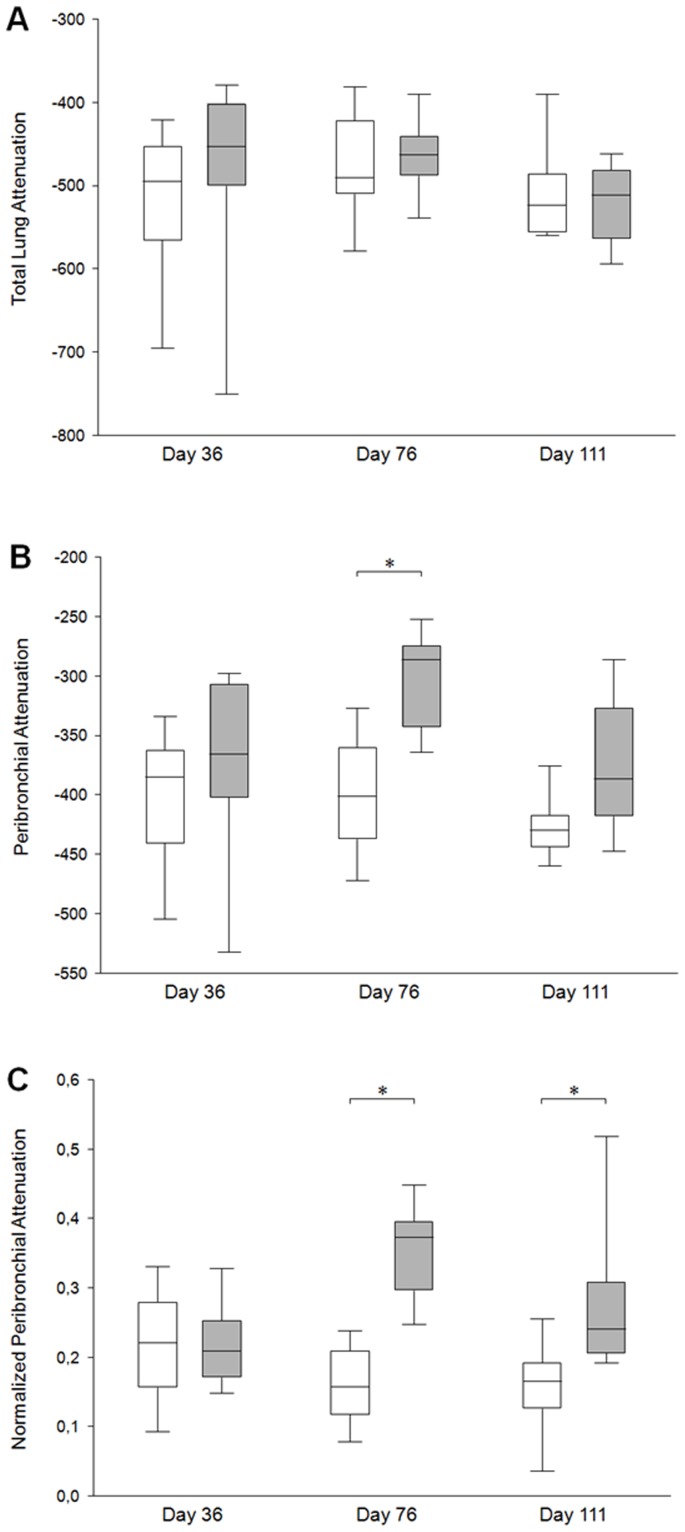
Comparison of micro-CT parameters. A) Total lung attenuation, B) peribronchial mean attenuation (PBA), and C) normalized PBA are presented for control (white box plots) and OVA-sensitized (grey box plots) mice at each endpoint. Box plots summarise medians with 25% and 75% interquartiles. Error bars represent 5th and 95th percentiles. *p<0.05 using Wilcoxon’s signed-rank tests between control and OVA.

**Figure 7:**
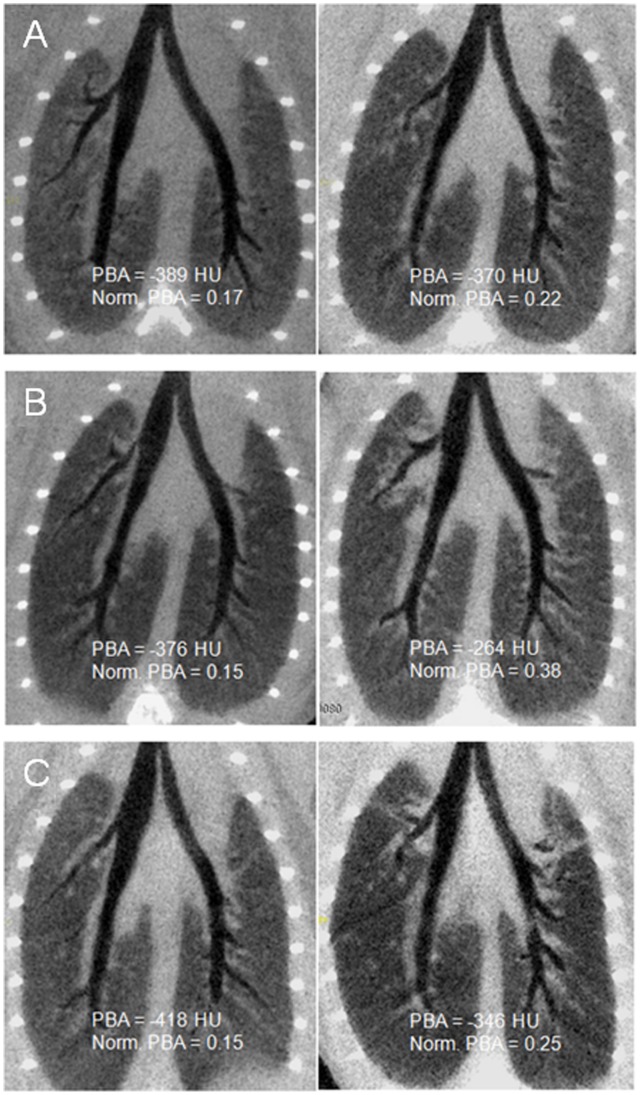
Typical coronal curved reformatted micro-CT images of the bronchial tree with numerical values of peribronchial mean attenuation (PBA) and normalized PBA. Images were obtained from control mice (left) and OVA-sensitized (right) at different endpoints: A) Day 36, B) Day 76 and C) Day 111.

**Figure 8:**
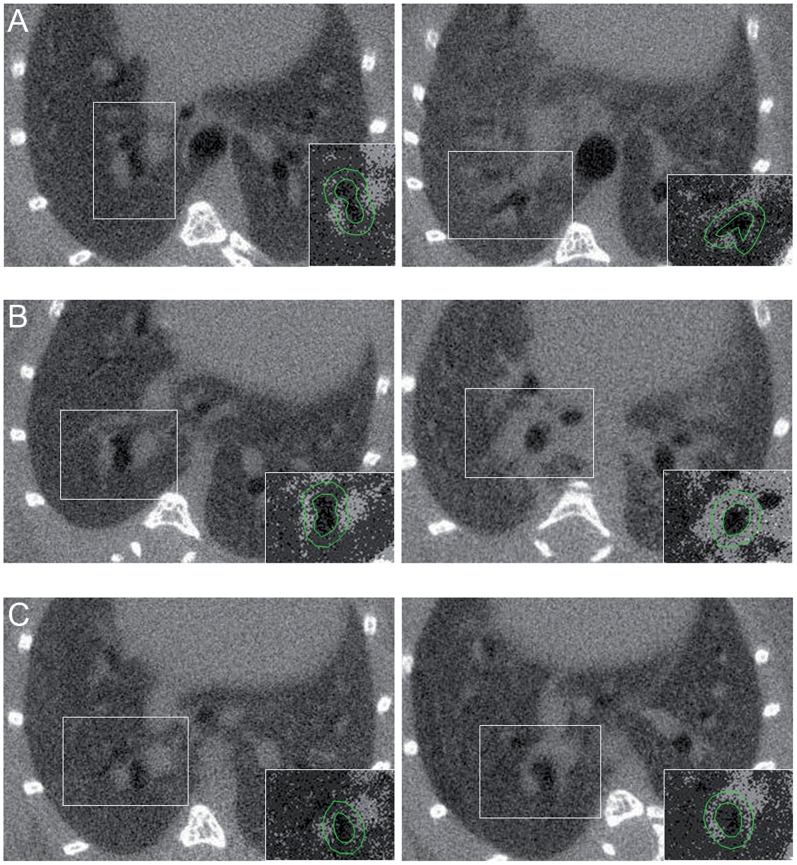
Typical axial native micro-CT images of control (left) and OVA-sensitized mice (right) at different endpoints: A) Day 36, B) Day 76 and C) Day 111. The insert at the right bottom of each panel corresponds to a selected part of a new image generated by normalizing each pixel attenuation value by the total lung attenuation value. The green circles delineating the lumen and the 8-voxels peribronchial atmosphere help to demonstrate the differences in normalized peribronchial attenuation between control and OVA-sensitized mice at day 76 and day 111.

**Table 2:** Correlation matrix between micro-CT bronchial parameters and plethysmographic, BAL and histological data.

	OVA sensitized mice (n = 26)	
	PBA	Normalized PBA
**Plethysmography**		
Penh ratio at endpoint	0.43 (0.03)	0.04 (0.841)
**BAL**		
Total cells (x 10^4^/mL)	0.37 (0.073)	0.45 (0.027)
% monocytes-macrophages	−0.53 (0.008)	−0.49 (0.015)
% neutrophils	0.26 (0.22)	0.38 (0.068)
% eosinophils	0.51 (0.012)	0.21 (0.326)
% lymphocytes	0.64 (0.001)	0.44 (0.032)
**Histological data**		
Peribronchial space area (µm^2^)	0.69 (<0.001)	0.59 (0.002)
Number of nucleated cells within the peribronchial	0.67 (<0.001)	0.56 (0.003)
space area (cell/µm^2^)		
Basement membranous thickness (µm)	0.18 (0.375)	0.27 (0.179)
Wall area (µm^2^/bronchus)	0.05 (0.813)	0.43 (0.03)
Bronchial muscle area (µm^2^/bronchus)	0.05 (0.792)	0.42 (0.034)
Peribronchial fibrosis (µm^2^)	0.29 (0.156)	0.53 (0.005)

Data are Spearman rank correlation coefficients. Data in parentheses are P-values.

BAL: bronchoalveolar lavage. PBA: peribronchial mean attenuation, Normalized PBA corresponds to 1– PBA/total lung mean attenuation.

### Micro-CT Imaging

The micro-CT procedure has been described previously [16]. Briefly, mice were anaesthetised, intubated, and connected to a dedicated ventilator for respiratory gating. The output signal of the ventilator allowed data acquisition to be triggered at the end of expiration. Images were acquired through a micro-CT system (eXplore Locus, GE Healthcare, London, ON, Canada) and were obtained in the absence of any contrast agent at 80 kV, 0.45 mA. The full acquisition lasted 17 min and the expected entrance dose was 0.26 Gy per scan. We obtained an average of 300 DICOM images with a 23-mm field of view and an isotropic 46×46×46 µm voxel size. Water, bone and air standards were placed in the chamber, in order to normalize the Hounsfield Units (HU) scale for each dataset acquisition. Volume datasets were exported to commercially available software (Myrian, Intrasense, Montpellier, France) in DICOM format, and information about the groups was blinded. All micro-CT images were analyzed in random order.

### Image Post-processing

From each micro-CT examination, 2 parameters were extracted using Myrian software:

the total lung mean attenuation (TLA) was automatically assessed using a volume-growing algorithm from bi-thresholded voxels (−900 to −100 HU).
the peribronchial mean attenuation (PBA) was assessed using a 3D semi-automatic method lasting 6–8 min and comprising 4 steps (Figure 2). The first step was to perform automatic segmentation of the bronchial lumen using a bi-threshold approach (−1024 to −900 HU). The second step applied an automatic three-dimensional morphologic dilatation tool to the volume of interest (VOI) obtained from the first step. This dilatation included the peribronchial space into the VOI. A 8-voxels dilatation level was found to be optimal to achieve the same peribronchial segmentation than with the previously validated manual method [16]. The third step consisted in creating a second segmentation VOI of the bronchial lumen overwriting the first VOI. The final step was to subtract the previous VOI (bronchial lumen only) from the initial VOI (bronchial lumen and peribronchial space). The trachea and the mediastinum were also manually subtracted. The resultant VOI displayed a mean attenuation value named PBA, which was recorded for further analysis. Then, normalized PBA was calculated as follow: 1-(PBA/TLA).

Finally, using the software MIPAV (Medical Image Processing Analysis and Visualization, National Institutes of Health, Bethesda, MD, USA), we have applied to selected axial images of each group, a mathematical algorithm that calculated for each pixel (with an attenuation value “x”) a new attenuation value “y”, using this formula: y = 1−(x/TLA).

### Bronchoalveolar Lavage

Bronchoalveolar lavage (BAL) was obtained at Day 37, 77 or 112 by cannulating the trachea with a 24-gauge catheter. The right lung was lavaged twice (each aliquot 0.3 ml; NaCl 0.9%). Total cell number was counted with a hemocytometer. Cytocentrifuge preparations (Cytospin 4, ThermoFisher Scientific, Courtaboeuf, France) were stained with Diff-Quik® (VWR International, Strasbourg, France), adapted from Giemsa-May-Grünwald stain. Differential cell counting was performed using standard morphological criteria in which 400 cells were analyzed by a blinded investigator using standard haematological criteria. Total leukocyte number, percentage of eosinophils, neutrophils, lymphocytes and macrophages were determined in each BAL fluid.

### Histology

Right and left lung tissue were dissected out, fixed with formaldehyde in inflation using intrapulmonary injection, and embedded in paraffin. Histological analysis was performed using 4-µm-thick lung slices stained with haematoxylin-eosin-safran and a modified Masson’s trichrome (half dilution of hematoxylin as compared to the standard staining procedure). Immunohistochemistry was performed using an anti-mouse alpha smooth muscle actin antibody clone 1A4 (Dako, Trappes, France). Formalin-fixed paraffin-embedded tissue sections were incubated in pretreatment buffer for antigen retrieval and were then reacted with a mouse anti-smooth muscle α-actin for 15 minutes. Immunoreaction was detected using Bond Polymer Refine Detection (Leica Microsystems, Wetzlar, Germany) on Bond TM max (A.Menarini Diagnostics, Firenze, Italy).

Several quantitative parameters were assessed using Quancoul software (Quant’Image, Bordeaux, France) at magnifications of 100× to 400× [18]. We measured the basal membrane thickness, the wall area, the bronchial smooth muscle area and the peribronchial area. We also assessed the area of fibrosis within the peribronchial space and the number of nucleated cells within the peribronchial space. All were measured on HES and actin-stained sections, except fibrosis which was quantified on modified Masson’s trichrome stain.

### Statistical Analysis

Values are expressed as the mean ± SEM, except those related to microCT for which the normality could not be rigorously established. The agreement between the semi-automatic and manual methods for PBA measurement was assessed in 10 datasets chosen at random using Bland-Altman analysis [19]. The manual method has been described previously in detail [16] and was based upon a two-dimensional analysis from multiplanar reformations.

For each group, the following parameters were compared between sensitized and control mice using Mann-Whitney-Wilcoxon rank sum test: weight at endpoint, Penh ratio, LR, micro-CT parameters, BAL results and histological data. Correlations between, on the one hand, PBA or normalized PBA, and, on the other hand, Penh ratio, BAL or histological data, were assessed using the Spearman rank correlation coefficients.

All analyses were performed using NCSS software (NCSS 2001, Kaysville, UT, USA) and results were considered statistically significant when P-values<0.05.

## Results

### Description of the Mouse Models of Asthma

From an initial set of 60 mice, 51 completed the study. Two mice died during the intubation procedure, 3 mice did not recover from anaesthesia following micro-CT, and 4 mice presented CT motion artefacts. Body weights were similar between control and OVA-sensitized mice at each endpoint. Table 1 displays experimental data from non invasive plethysmography, bronchoalveolar lavage, and histological parameters for each group. Sensitized mice from group A (Days 35–37) exhibited features of BHR to methacholine, as assessed by a significant increase in Penh ratio, characteristics of airway inflammation, as assessed by the increased percentage of both eosinophils and lymphocytes within the BAL fluid, but no evidence of bronchial remodeling as compared to control animals (Table 1, Figure 3A). Sensitized mice from group B (Days 75–77) also exhibited features of BHR to methacholine assessed by non invasive plethysmography (Table 1, Figure 4A). Similar results were obtained using invasive plethysmography (Figure 4). These mice also displayed more pronounced characteristics of airway inflammation, and additionally patterns of bronchial remodeling as assessed by the increased basal membrane thickness, wall area and bronchial smooth muscle area (Table 1, Figure 3B). In contrast, sensitized mice from group C (Days 110–112) did not show any evidence of BHR or airway inflammation but a significant increase in all previous markers of airway remodeling (Table 1, Figure 3C).

### Validation of a Semi-automatic Method for PBA Assessment

PBA measurements obtained with the semi-automatic method showed a good agreement with PBA values obtained with the manual method (Figure 5). The Pearson’s correlation coefficient was 0.963. The intraclass correlation coefficient was 0.933. The measurement error between the two methods was 19 HU. Standard deviations of measurements did not correlate with mean values.

### Comparisons of Micro-CT Parameters

There was no difference in TLA between sensitized and control mice whatever the group (Figure 6A). Conversely, PBA was significantly higher in sensitized mice but only from the group B exhibiting both inflammation and remodeling (Figure 6B). However, normalized PBA was significantly higher in sensitized mice from both groups B and C (Figure 6C). Indeed, in group B, medians of normalized PBA increased from 0.16 to 0.37 (p<0.001), and, in group C, from 0.17 to 0.24 (p = 0.009) in control and sensitized mice, respectively. Typical micro-CT images from each group are illustrated (Figure 7). Since these original images did not reflect normalized PBA, a new set of images has been generated by normalizing each pixel attenuation value by the mean lung attenuation value (Figure 8).

### Correlations between Micro-CT Bronchial Parameters, Penh Ratio, BAL and Histological Parameters

A correlation matrix has been built within the sensitized mice (Table 2). Penh ratio was significantly correlated with PBA but was not correlated with normalized PBA. With regards to inflammatory markers from the bronchoalveolar lavage fluid, both PBA and normalized PBA were significantly correlated with the percentage of lymphocytes. Both were also correlated with the peribronchial space and the number of nucleated cells within the peribronchial space. Finally, only normalized PBA was significantly correlated with remodeling parameters such as bronchial wall area, smooth muscle area and peribronchial fibrosis. The higher the normalized PBA, the higher the bronchial smooth muscle remodeling was.

## Discussion

Taken together, these results demonstrate that, using a flexible model of murine asthma, normalized PBA extracted from micro-CT examinations in living mice, can predict the presence of airway remodeling. The peribronchial attenuation value normalized by the total lung attenuation value was increased in mice exhibiting remodeling, was unchanged in mice exhibiting inflammation only, and was the best micro-CT parameter correlated with remodeling markers.

In this study, we paid a special attention to build flexible challenge protocols based upon different endpoints which reproduced 3 features of human asthma (*i.e*. inflammation only, inflammation and remodeling, and remodeling only), although the latter remains theoretical, since inflammatory cells are still present in fixed airways obstruction [20]. Particularly, eosinophilic inflammation was observed in groups A and B only, while the main markers of remodeling, *i.e.* increased bronchial smooth muscle size and peribronchial fibrosis, were observed in groups B (day 75) and C (Day 110) only. The use of Penh to assess BHR in mice deserves a specific comment. Indeed, Penh does not represent the airway resistance per se [21] and it may vary according to the respiratory rate and/or experimental conditions [22]. For instance, Penh is not accurate in C57BL6 mice [23]. However, in our study, both Penh and LR ratios were similarly increased in OVA-sensitized mice as compared to control mice, which is in agreement with earlier studies performed in Balb/C mice [23]. Moreover, invasive plethysmography cannot be performed longitudinally. BHR is one of the characteristics of asthma but the exact contribution of inflammation or remodeling remains undetermined [24]. In our study, BHR assessed by the Penh ratio was only observed in mice exhibiting inflammation either alone or with remodeling. In small animals, even if clear model-dependent differences have been shown [25], Penh ratio has been shown to be mainly related to eosinophilic inflammation in Balb/C mice [26], which is consistent with our results.

So far, to the best of our knowledge, there was no reported *in vivo* method able to assess bronchial remodeling noninvasively. By contrast, airway inflammation can be assessed through exhaled nitric oxide or induced sputum [27], [28]. In the present study, we demonstrated that micro-CT can quantify remodeling non-invasively in sensitized mice. However, PBA and normalized PBA were also correlated with some parameters of bronchial inflammation. These results can be partly explained by the close relationship between inflammation and remodeling [29], [30], which is likely to entail potential cross-correlations. Our 3 endpoints protocol allowed us to demonstrate the absence of any significant difference in micro-CT parameters between sensitized and control mice from group A, thereby suggesting that the sole inflammation has no influence on PBA or normalized PBA. In the absence of normalization by the lung attenuation value, PBA appeared to be less specific to remodeling and only increased in mice exhibiting both inflammation and remodeling, which is consistent with our previous study [16]. Such a result can be explained by the fact that the region of interest of the PBA encompassed lung parenchyma beyond the bronchial wall, thereby including inflammatory cells located outside the bronchial wall (Figs. 3A and 3B). Indeed, in mice as well as in humans, remodeling is only located within the bronchial wall whereas inflammation occurs not only in bronchi but also in the distal lung parenchyma [17], [31]. Therefore, the normalization by the whole lung attenuation might have withdrawn inflammation from the PBA value.

Our results suggest that micro-CT could be considered for monitoring remodeling when testing new drugs targeting remodeling in longitudinal studies. Micro-CT could also be used as a complement of histology since our technique allows a three-dimensional and comprehensive assessment of remodeling, while histology only provides two-dimensional information from small samples. The 3D semi-automatic method, we validated here, is less time-consuming (8 min vs. 40 min) than the manual method [16] since it requires no manual drawing. Of note, micro-CT examinations were performed after endotracheal intubation. Intubating a mouse is a subtle technique requiring a training phase and may induce injury if improperly done. Free-breathing techniques have been developed to avoid intubation [32], [33], however they cannot reach the same quality of gating than those under mechanical ventilation. To avoid any confounding effect related to repeated anaesthesia, tracheal intubation, or radiation exposure, we did not study a unique cohort of mice at three different time points. Likewise, age-matched control mice were necessary to avoid potential confounding effects due to age-related changes.

Potential applications in humans are also conceivable. Even if molecular imaging is thought to play a crucial role in a near future by targeting specific proteins or receptors involved in asthma [34], multidetector CT might be an easier cost-effective tool, and is immediately available. In COPD patients, bronchial wall attenuation has been recently shown to be increased as compared to control subjects, and significantly correlated to functional obstructive parameters [35]–[37]. Thus, the peribronchial attenuation might be considered as a potential translational concept. Our results in mice should open the way to further studies in humans, aimed at identifying CT markers of asthma.

To conclude, a non-invasive assessment of bronchial remodeling in asthmatic mice is feasible using *in vivo* respiratory-gated micro-CT. The peribronchial attenuation value normalized by the total lung attenuation value appears to be the most reliable marker of remodeling. It may help evaluate new drugs targeting airway remodeling in pre-clinical and clinical studies.
